# Risk of Cardiac Adverse Events in Patients Treated With Immune Checkpoint Inhibitor Regimens: A Systematic Review and Meta-Analysis

**DOI:** 10.3389/fonc.2021.645245

**Published:** 2021-05-27

**Authors:** Jiexuan Hu, Ruyue Tian, Yingjie Ma, Hongchao Zhen, Xiao Ma, Qiang Su, Bangwei Cao

**Affiliations:** ^1^ Department of Oncology, Beijing Friendship Hospital, Capital Medical University, Beijing, China; ^2^ Department of Ultrasound, Aero Space Central Hospital, Beijing, China

**Keywords:** cardiovascular toxicity, PD-1/PD-L1 inhibitors, chemotherapy, solid tumors, meta-analysis

## Abstract

**Background:**

We performed a systematic review and meta-analysis to evaluate the risks of cardiac adverse events in solid tumor patients treated with monotherapy of immune checkpoint inhibitors (ICIs) or combined therapy of ICIs plus chemotherapy.

**Methods:**

Eligible studies were selected through the following databases: PubMed, Embase and clinical trials (https://clinicaltrials.gov.) and included phase III/IV randomized controlled trials (RCTs) involving patients with the solid tumor treated with ICIs. The data was analyzed by using Review Manager (version5.3), Stata (version 15.1).

**Results:**

Among 2,551 studies, 25 clinical trials including 20,244 patients were qualified for the meta-analysis. Compared with PD-1 inhibitor (nivolumab) or CTLA-4 inhibitor (ipilimumab), PD-1 inhibitor (nivolumab) plus CTLA-4 inhibitor (ipilimumab) combined therapy showed significant increase in grade 5 arrhythmology (OR 3.90, 95% CI: 1.08–14.06, p = 0.603). PD-1 inhibitor plus chemotherapy show significant increase in grades 1–5 myocardial disease (OR 5.09, 95% CI: 1.11–23.32, p = 1.000). Compared with chemotherapy, PD-1 inhibitor (nivolumab) or CTLA-4 inhibitor (ipilimumab), PD-1 inhibitor (nivolumab) plus CTLA-4 inhibitor (ipilimumab) combined therapy show significant increase in grades 1–5 arrhythmology (OR 2.49, 95% CI: 1.30–4.78, p = 0.289).

**Conclusions:**

Our meta-analysis demonstrated that PD-1 inhibitor plus CTLA-4 inhibitor can result in a higher risk of grade 5 arrhythmology in comparison with PD-1/CTLA-4 inhibitor alone, and a higher risk of grade 5 arrhythmology in comparison with chemotherapy. PD-1 inhibitor plus chemotherapy treatment could increase the risk of all-grade myocardial disease compared with chemotherapy. However, in most cases, there was no significant increase of risks of cardiovascular toxicity in PD-1/PD-L1 inhibitor monotherapy or PD-1/PD-L1 inhibitor plus chemotherapy compared with chemotherapy alone.

## Introduction

The progression of immunotherapy have considerably changed cancer treatment, and improved the clinical prognosis of many cancer patients. Researches showed that monoclonal antibodies against cytotoxic T-lymphocyte-associated protein-4 (CTLA-4) and programmed cell death protein 1 (PD-1)/programmed cell death protein 1 ligand 1 (PD-L1) are beneficial for solid tumor patients during treatment (1). Thus far, a list of ICIs have been approved by Food and Drug Administration(FDA), including PD-L1 inhibitors (atezolizumab, durvalumab, and avelumab), CTLA4 inhibitor (ipilimumab), and PD-1 inhibitors (nivolumab and pembrolizumab) etc., which were applied to treatable tumors including melanoma, Hodgkin’s lymphoma, non-small-cell lung cancer, renal cell carcinoma, bladder, head and neck cancer, liver, gastric cancer and microsatellite instability high or DNA mismatch repair-deficient colorectal cancer (2), Nevertheless, since immune checkpoints also play pivotal roles in maintaining homeostasis of normal tissues, their therapeutic blockade can unavoidably cause side effects to the patients, termed as immune-related adverse events (irAEs). IrAEs have been shown to affect many systems and cause lesions of differential severity, like pneumonitis and pancreatitis. Although relatively less, cardiotoxicity has also been found following cancer-related treatments such as chemotherapy and targeted therapy, which sometimes, like the myocarditis, can be fatal. However, irAEs concerning cardiovascular toxicity has not been well recognized or reported (3). In the current study, the risks of immune-related cardiac toxicity in cancer patients treated with ICIs were systematically evaluated, which was supposed to help better understand the cardiac harmness of ICIs and provide a reference for the rational use and safety evaluation of ICIs in clinical practice.

## Methods

### Search Methods and Study Selection

The systematic review with meta-analysis was conducted according to the guidelines of the Cochrane Handbook for Systematic Reviews of Interventions, and reported according to the PRISMA Statement (4).

We searched the literatures related to the efficacy of immunosuppressive agents in solid tumors from the following databases: PubMed, Embase and clinicaltrials (https://clinicaltrials.gov) up to Oct, 2020. The medical subject heading (MeSH) terms included for searching the relevant studies containing the following keywords and terms: one term that refers to cancer (neoplasm, carcinoma, cancer, or tumor), one term indicating the ICIs (anti-CTLA-4, anti-PD-1, ipilimumab, tremelimumab, nivolumab, pembrolizumab, atezolizumab, durvalumab, or avelumab), and one term related to randomized controlled trials, connected by “and”. After the initial search, we further selected studies according to the following criteria: (1) phase III/IV RCTs with primary endpoints such as overall survival (OS), progression-free survival (PFS), or objective response rate (ORR); (2) histologically confirmed solid cancers such as lung cancer, and others; (3) containing the information of ICIs and cardiotoxicity; and (4) sharing some similarity in experimental method across different studies. Moreover, studies were excluded if they were (1) reviews, duplicate reports, letters, unfinished studies, or conference reports; (2) studies where cardiotoxicity cannot be confirmed due to insufficient data; (3) papers in languages other than English; (4) studies conducted with cell lines, animal models, or on nonsolid cancers; (5) studies whose experimental method was substantially different from other selected RCTs; and (6) RCTs in phase I/II. This study was performed according to the Preferred Reporting Items for Systematic Reviews and Meta-analyses (PRISMA) guideline.

### Data Extraction

Two reviewers (JXH and RYT) independently searched all the relevant studies and read the titles, abstracts, and full texts of the identified studies. The eligibility of the extracted studies was assessed using the PICO (patient, intervention, comparison and outcome) chart. Two reviewers extracted the following information from the selected study: year of publication, name of the journal, the last name of the first author, treatment arms, the primary endpoint, type of underlying solid tumor, number of patients in the ICIs treatment groups, number of patients in control groups, number of patients bearing pneumonitis or pneumonia of all-grade (grades 1–5), high-grade (grades 3–5), and death. Disagreements in the assessment were resolved *via* discussion with the third reviewer (QS).

Cardiac toxicity related AEs are of a large variety yet the incidence of each type is small. We classified irAEs into six major categories (5):

1. arrhythmology, namely atrial fibrillation, atrial flutter, atrial tachycardia, atrioventricular block, arrhythmia supraventricular, atrioventricular block complete, bradycardia, bifascicular block, sinus bradycardia, sinus tachycardia, supraventricular tachycardia, tachycardia, ventricular arrhythmia, ventricular tachycardia, ventricular fibrillation;
2. cardiac failure, which refers to cardiac failure, acute cardiac failure, chronic cardiac failure, congestive, cardiopulmonary failure, right ventricular failure;
3. coronary artery disease, such as arteriospasm coronary, acute coronary syndromes, angina unstable, angina pectoris, acute myocardial infarction, myocardial infarction, myocardial ischemia;
4. pericardial disease, including cardiac tamponade, pericardial effusion, pericarditis);
5. cardiac arrest, for example cardio-respiratory arrest;
6. myocardial disease, comprising of autoimmune myocarditis, eosinophilic myocarditis, myocarditis).

### Data Analysis

We used pooled odds ratios (ORs) with 95% CIs to evaluate the risk of cardiac adverse events of ICIs. We mainly collect the drugs used in clinical trials, major types of adverse cardiac reactions, number of patients in experimental and control groups. Heterogeneity was evaluated using the Q test and Higgin’s and Thompson’s I2 (I2) statistics. Defined as the percentage of variations in the effect sizes that is not the cause of sampling errors, I2 statistics is robust and not sensitive to the number of studies under analysis. We consider I2 values greater than 50% as moderate to high heterogeneity. We used random-effects model (DerSimonian and Laird method) if the I2 value was more than 50%, and used Mantel Haenszel Model to assess the heterogeneity. P <0.05 was considered statistically significant. The quality of the included studies was assessed by Cochrane risk of bias tool. We performed subgroup analysis (based on drugs, treatments and irAEs) and sensitivity analysis to find the source of heterogeneity. Specifically, we sequentially removed individual studies one by one to evaluate the stability of the results. The incidence of each type Cardiac toxicity related AEs is small, we used the continuous correction method to correct our data. Finally, we applied the Begger test, Egger test and funnel plot to assess publication bias. All analyses were performed using Review Manager (version 5.3), Stata (version 15.1), RStudio.

## Results

### Eligible Studies and Characteristics

**Figure 1:**
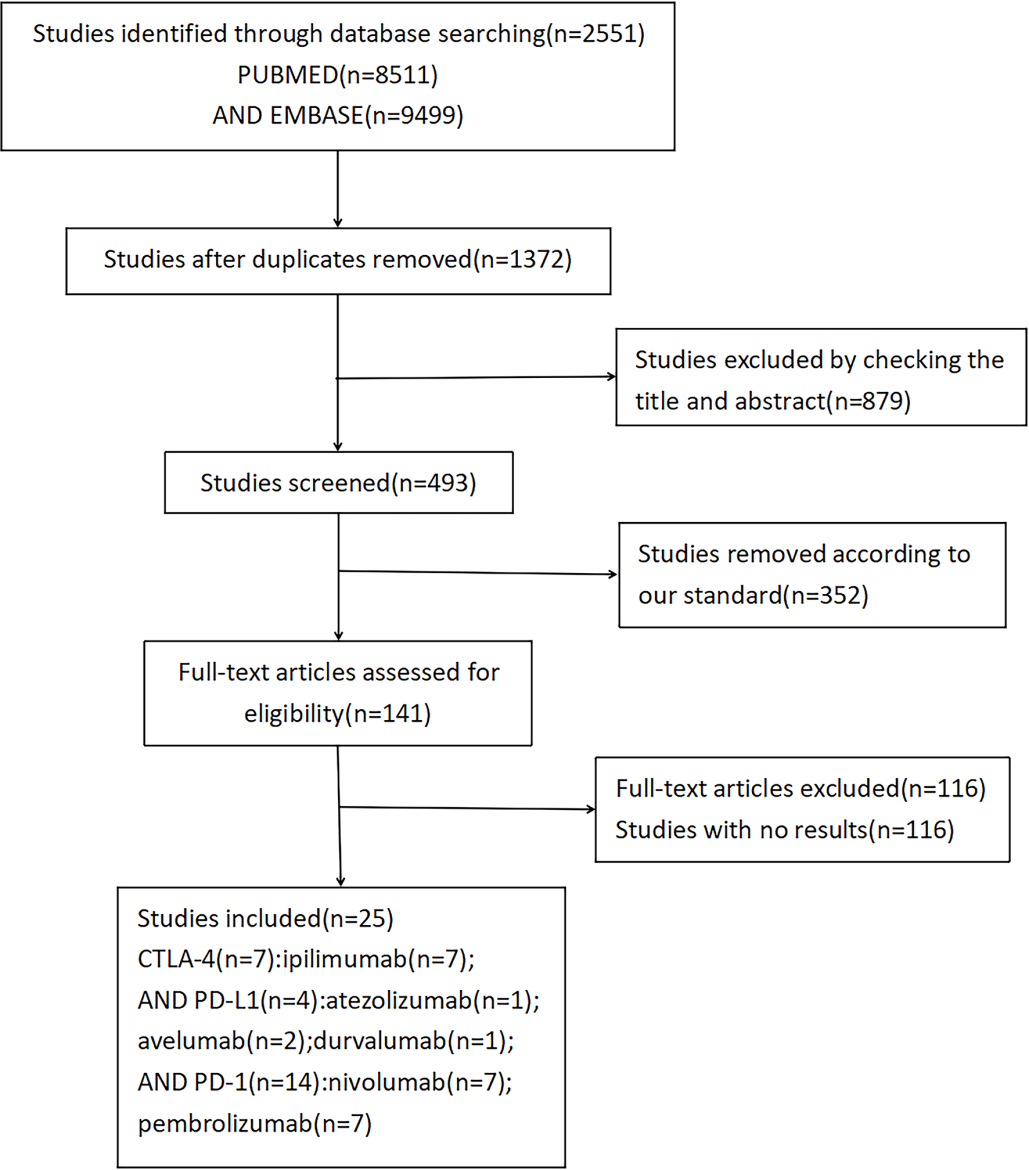
Flowchart depicting the studies selection process.

Using the search terminology, we initially identified 2,551 studies from the three databases. Among those studies, 25 RCTs met our rigorous inclusion criteria (**Table 1**) (6–30). The detailed research flow chart is shown in **Figure 1**. All 25 trials evaluated and compared the side effects of ICI with other control treatments. A total of 20,244 patients were included in the 25 trials, including seven studies related to CTLA-4 (ipilimumab: seven cohorts, n = 8,134), four studies related to PD-L1 (atezolizumab: one cohort, n = 403; avelumab: two cohorts, n = 1,358; durvalumab: one cohort, n = 709), 14 studies related to PD-1 (nivolumab: seven cohorts, n = 5,810; pembrolizumab: seven cohorts, n = 3,830) and 11 studies compared combined therapy of chemotherapy plus ICIs with chemotherapy. In terms of patients’ tumor type, four trials evaluated non-small-cell lung cancer (NSCLC), one trial evaluated small-cell lung cancer, 10 evaluated melanoma, two studies evaluated advanced renal-cell carcinoma, two trials evaluated multiple myeloma, one trial evaluated advanced urothelial carcinoma, one evaluated prostate cancer, and one trial evaluated ovarian cancer.

**Table 1:** List of the 25 studies included in this meta-analysis.

Study [year]	Drug	Histology	Endpoint	Treatment arms	Patients	Arrhythmology(grades 1–5, n)	Cardiac failure (grades 1–5, n)	Coronary artery disease (grades 1–5, n)	Pericardial disease (grades 1–5, n)	Cardiac arrest(grades 1–5, n)	Myocardial disease (grades 1–5, n)
Nishio (6)	PD-L1	Small Cell Lung Carcinoma	PFS	Arm1: Atezolizumab + Carboplatin + Etoposide	403 (198/196)	2/4	0/2	NA	2/1	NA	NA
				Arm2: Placebo + Carboplatin + Etoposide							
Pujade-Lauraine (7)	PD-L1	Platinum-resistant/refractory ovarian cancer	OS	Arm1: Avelumab 10 mg/kg q2w	566 (187/182/177)	1/1/0	NA	0/1/0	NA	NA	NA
				Arm2: Avelumab 10 mg/kg q2w plus PLD 40 mg/m2 q4w							
				Arm3: PLD 40 mg/m2 q4w							
Barlesi (8)	PD-L1	Non-Small-Cell Lung	OS	Arm1: Avelumab 10 mg/kg q2w	792 (393/365)	3/1	2/1	3/2	4/0	2/0	3/0
				Arm2: Docetaxel 75 mg/m2 q3w							
Antonia (9)	PD-L1	Non–Small-Cell Lung	PFS	Arm1: Durvalumab 10 mg/kg q2w	709 (473/236)	6/1	6/0	9/0	2/0	2/1	1/1
				Arm2: Placebo							
Govindan (10)	CTLA-4	Advanced Squamous Non–Small-Cell Lung Cancer	OS	Arm1: Ipilimumab 10 mg/kg q3w	1,289 (475/473)	10/12	2/5	2/1	0/4	7/4	NA
				Arm2: Placebo							
Ascierto (11)	CTLA-4	Unresectable or metastatic melanoma	OS	Arm1: Ipilimumab 10 mg/kg q3w vs	831 (364/362)	6/0	2/0	0/2	2/1	1/1	NA
				Arm2: Ipilimumab 3 mg/kg q3w							
Reck (12)	CTLA-4	Extensive-Stage Small-Cell Lung Cancer	OS	Arm1: Ipilimumab 10 mg/kg q3w + Etoposide + Cisplatin/Carboplatin	1,351 (562/561)	5/3	5/8	5/5	2/3	2/0	NA
				Arm2: Placebo matching Ipilimumab + Etoposide + Cisplatin/Carboplatin							
Eggermont (13)	CTLA-4	High-risk stage III melanoma	OS	Arm1: Ipilimumab 10 mg/kg q4w Arm2: Placebo	1211 (471/474)	4/4	1/1	2/1	NA	0/1	1/0
Kwon (14)	CTLA-4	Metastatic castration-resistant prostate cancer	OS	Arm1: Ipilimumab 10 mg/kg q4w	988 (393/396)	8/2	4/3	4/2	1/0	4/2	NA
				Arm2: placebo							
Robert (15)	CTLA-4	Previously Untreated Metastatic Melanoma	OS	Arm1: Ipilimumab 10 mg/kg q3w+Dacarbazine 850 mg/kg q3w	681 (247/251)	2/3	NA	1/2	0/1	NA	NA
				Arm2: Dacarbazine 850 mg/m^2 q3w + placebo							
Hodi (16)	CTLA-4	Metastatic Melanoma	OS	Arm1: ipilimumab + Placebo	1,783 (131/380/382)	0/2/1	2/3/0	1/0/0	0/2/0	NA	NA
				Arm2: ipilimumab + Melanoma Peptide Vaccine							
				Arm3: Melanoma Peptide Vaccine + Placebo							
Burtness (17)	PD-1	Recurrent or Metastatic Squamous Cell Cancer of the Head and Neck	PFS	Arm1: Pembrolizumab 200 mg each 3-week	882 (301/281/300)	1/2/6	1/2/0	4/5/4	NA	0/2/0	0/2/0
				Arm2: Pembrolizumab + Chemotherapy							
				Arm3: Cetuximab + Chemotherapy							
Mateos (18)	PD-1	Refractory or Relapsed and Refractory Multiple Myeloma	PFS	Arm1: Pembrolizumab + Pomalidomide + Dexamethasone	251 (122/123)	3/1	1/1	2/0	1/0	NA	2/0
				Arm2: Pomalidomide + Dexamethasone							
Long (19)	PD-1	Unresectable or Metastatic Melanoma	PFS	Arm1: Pembrolizumab + Epacadostat	706 (353/352)	2/1	NA	0/1	NA	NA	2/0
				Arm2: Pembrolizumab + Placebo							
Usmani (20)	PD-1	Newly Diagnosed Treatment Naive Multiple Myeloma	PFS	Arm1: Pembrolizumab + Lenalidomide + Dexamethasone	310 (154 148)	6/2	2/2	1/3	NA	5/2	2/0
				Arm2: Lenalidomide + Dexamethasone							
Haddad (21)	PD-1	Recurrent or Metastatic Head and Neck Carcinoma	OS	Arm1: Nivolumab	361 (236 111)	1/3	2/0	1/0	1/0	1/1	NA
				Arm2: Cetuximab/Methotrexate/Docetaxel							
Lebbé (22)	PD-1	Melanoma	ORR	Arm1: Nivolumab 3 mg/kg IV + Ipilimumab 1 mg/kg	481 (180 178 27)	1/1/0	1/0/0	3/0/0	NA	NA	2/0/0
				Arm2: Ipilimumab 3 mg/kg IV + Nivolumab 1 mg/kg IV							
				Arm3: Nivolumab 6 mg/kg IV + Ipilimumab 1 mg/kg							
Hod (23)	PD-1	Unresectable or Metastatic Melanoma	PFS	Arm 1: Nivolumab + Placebo for Ipilimumab + Placebo for Nivolumab	1,296 (313 313 311)	5/22/12	0/1/3	1/1/1	0/1/0	0/0/1	1/0/0
				Arm 2: Nivolumab + Ipilimumab + Placebo for Nivolumab							
				Arm 3: Ipilimumab + Placebo for Nivolumab							
Motzer (24)	PD-1	Advanced Renal-Cell Carcinoma	ORR	Arm1: Nivolumab 3 mg/kg + Ipilimumab 1 mg/kg	1,390 (547 535)	3/2	2/5	9/4	NA	4/2	1/0
				Arm2: Sunitinib 50 mg							
Bellmunt (25)	PD-1	Advanced Urothelial Carcinoma	OS	Arm1: Pembrolizumab 200 mg q3w	542 (255 266)	2/3	NA	0/1	1/0	NA	NA
				Arm2: paclitaxel 175 mg/m^2^ or docetaxel 75 mg/m^2^ IV or vinflunine 320 mg/m^2^ q3w							
Reck (26)	PD-1	PD-L1–Positive Non–Small-Cell Lung Cancer	PFS	Arm1: Pembrolizumab 200 mg q3w	305 (154 150)	1/5	1/3	0/3	2/3	1/2	NA
				Arm2: SOC Chemotherapy							
Motzer (27)	PD-1	Advanced Renal-Cell Carcinoma	OS	Arm1: Nivolumab 3 mg/kg q2w	1,068 (406 397)	4/4	3/4	1/1/0	0/1	0/2	NA
				Arm2: Everolimus 10 mg tablets by mouth daily							
Weber (28)	PD-1	Advanced melanoma who progressed after anti-CTLA-4 treatment	ORR	Arm1: Nivolumab 3 mg/kg q2w	631 (268 102)	8/0	1/0	2/0	1/0	4//0	NA
				Arm2: Dacarbazine 1,000 mg/m^2^ q3w or paclitaxel 175 mg/m² q3w							
Robert (29)	PD-1	Advanced Melanoma	PFS	Arm1: Pembrolizumab 10 mg/kg q2w	834 (256 278 277)	1/1/1	1/5/0	1/2/1	1/0/1	0/0/1	NA
				Arm2: Pembrolizumab 10 mg/kg q3w							
				Arm3: Ipilimumab 3 mg/kg q3w							
Robert (30)	PD-1	Previously Untreated Melanoma without BRAF Mutation	OS	Arm1: Nivolumab 3 mg/kg q2w	583 (206 205)	3/1	1/0	NA	NA	NA	NA
				Arm2: Dacarbazine 1,000 mg/m^2^ q3w							

NA, Not available; PFS, Progression-Free Survival; OS, Overall Survival; ORR, Objective Response Rate.

### Risk of Cardiotoxicity in Patients With PD-1/PD-L1 and CTLA-4 Inhibitors

**Figure 2:**
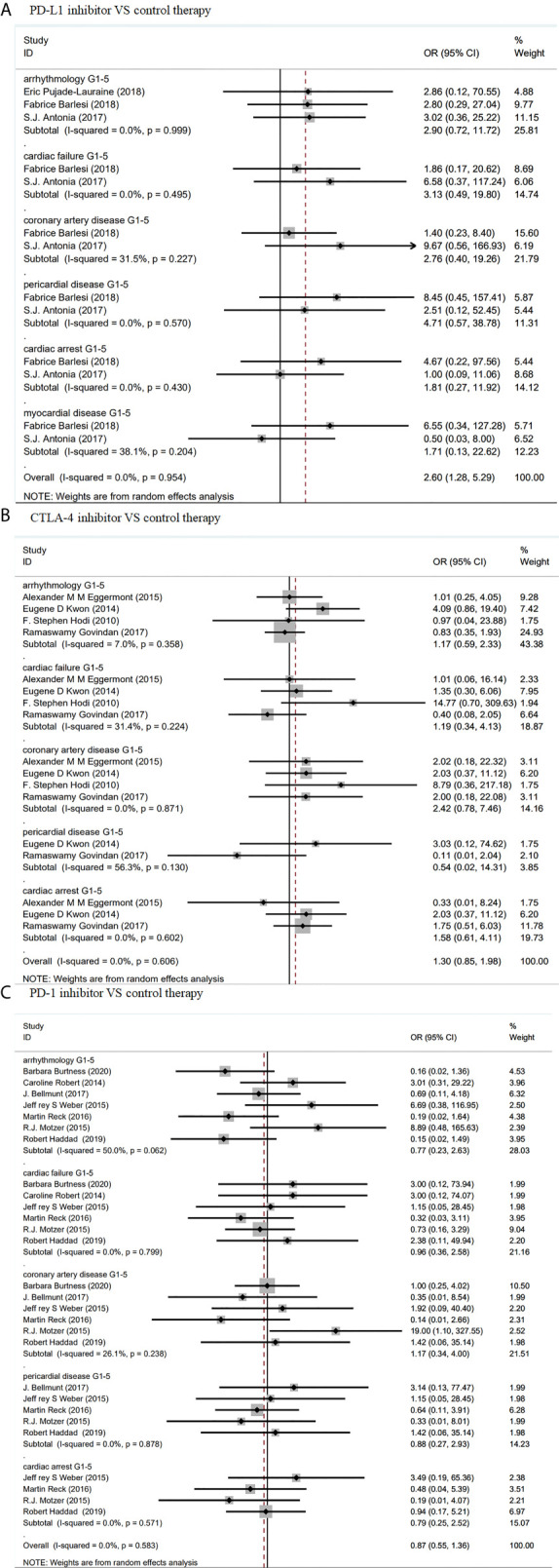
Forest plot analysis of cardiotoxicity in patients treated with ICIS compared with control therapy **(A)** Forest plot analysis of cardiotoxicity in patients treated with PD-L1 inhibitor compared with control therapy: G1–5, grades 1–5. **(B)** Forest plot analysis of cardiotoxicity in patients treated with CTLA-4 inhibitor compared with control therapy: G1–5, grades 1–5. **(C)** Forest plot analysis of cardiotoxicity in patients treated with PD-1 inhibitor compared with control therapy, G1–5: grades 1–5.

As shown in **Figure 2**, compared with control treatments, there was no significant increase of risk of cardiotoxicity in PD-L1 inhibitors. Specifically, the odd ratios (ORs) of different cardiotoxicity were as follows: grades 1–5 arrhythmology, 2.90 (95% CI: 0.72–11.72, p = 0.999); grades 1–5 cardiac failure, 3.13, (95% CI: 0.49–19.8, p = 0.495); grades 1–5 coronary artery, 2.76 (95% CI: 0.40–19.26, p = 0.227); grades 1–5 pericardial disease, 4.71 (95% CI: 0.57–38.78, p = 0.570), grades 1–5 cardiac arrest, 1.81 (95% CI: 0.27–11.92, p = 0.430) and myocardial disease, 1.71 (95% CI: 0.13–22.62, p = 0.204). Similar results were found in patients with CTLA-4/PD-1 inhibitors (**Figure 2**).

### Risk of Serious Adverse Events of Cardiotoxicity in ICIs Therapy

Since the studies we selected did not explicitly present cardiac-related adverse events, we mainly extracted the information through the https://clinicaltrials.gov. Cardiac-related adverse events were generally recorded as serious adverse events, which were put into four grades according to the severity in our analysis. Death was also considered to be cardiac-related serious adverse events here.

**Figure 3:**
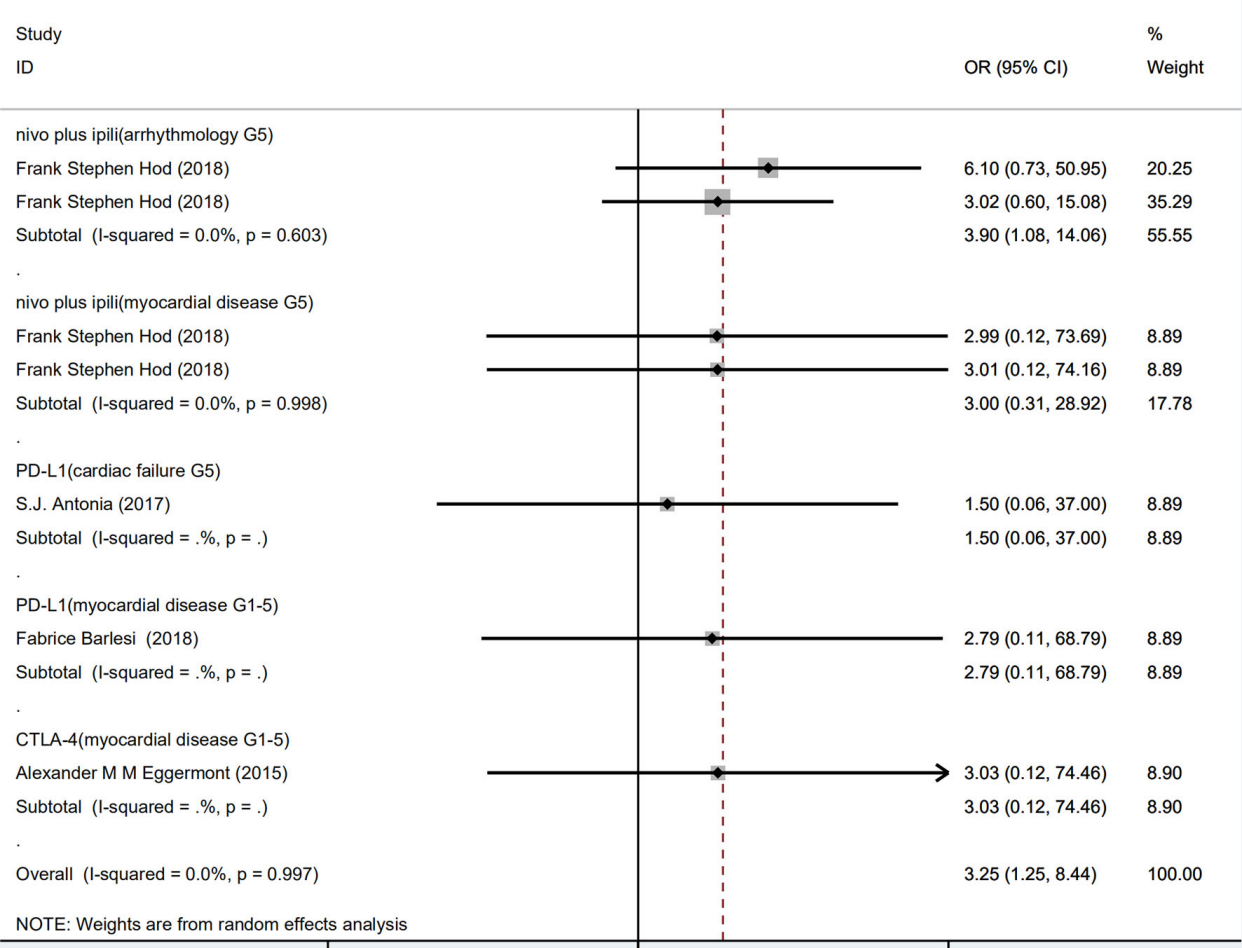
Forest plot analysis of serious adverse events: nivo plus ipili, PD-1 inhibitor (nivolumab) plus CTLA-4 inhibitor (ipilimumab) combination therapies; PD-L1, PD-L1 inhibitor(avelumab) compared with chemotherapy; CTLA-4, CTLA-4 inhibitor (ipilimumab) compared with chemotherapy; G5, grade 5.

As shown in **Figure 3**, compared with PD-1 inhibitor (nivolumab) or CTLA-4 inhibitor (ipilimumab) monotherapy, PD-1 inhibitor (nivolumab) plus CTLA-4 inhibitor (ipilimumab) combined therapy showed significant increase in grade 5 arrhythmology (OR 3.90, 95% CI: 1.08–14.06, p = 0.603), but not in grade 5 myocardial disease (OR 3.00, 95% CI: 0.31–28.92, p = 0.998).

There was no significant increase of risk of PD-L1 in grade 5 cardiac failure (OR 1.50, 95% CI: 0.06–37.00) and grade 5 myocardial disease (OR 2.79, 95% CI: 0.11–68.79), compared with chemotherapy. Similarly, CTLA-4 inhibitor did not significantly increase risk of grade 5 myocardial disease (OR 3.03, 95% CI: 0.12–74.46).

### Risk of Cardiotoxicity in Combined Therapies

**Figure 4:**
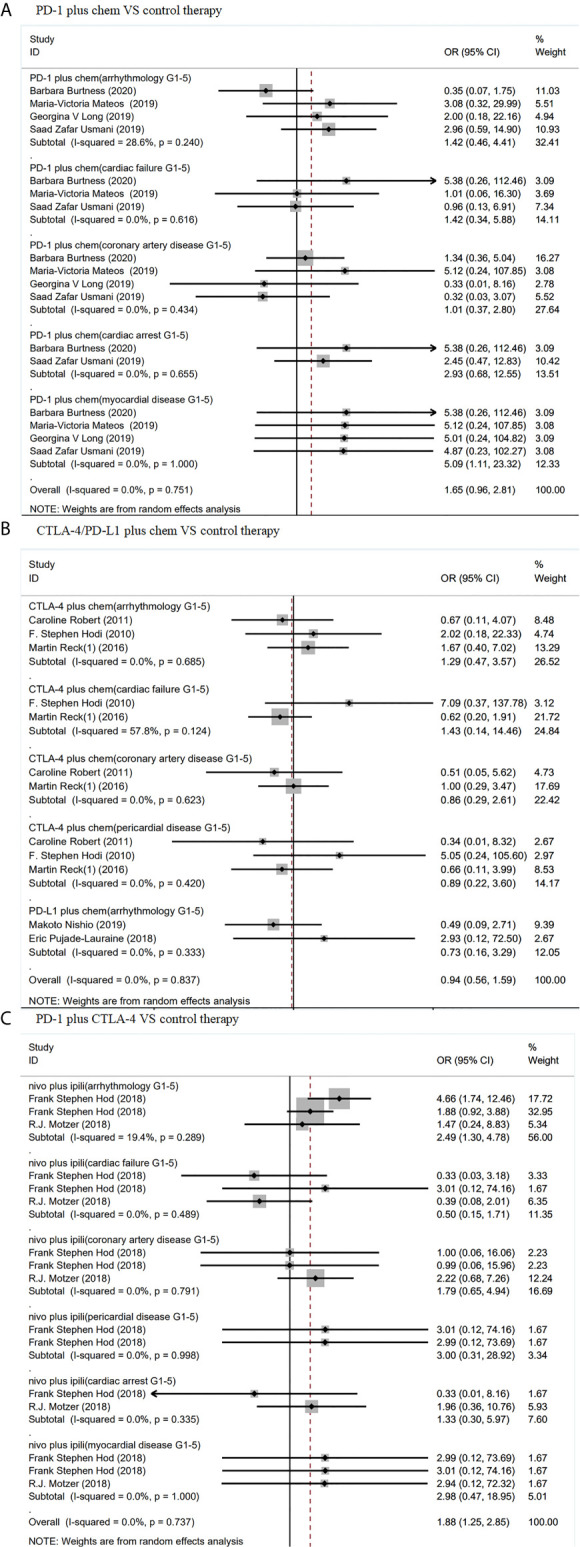
Forest plot analysis of cardiotoxicity in patients treated with combination therapy compared with control therapy **(A)** Forest plot analysis of cardiotoxicity in patients treated with PD-1 inhibitor plus chemotherapy combination therapy compared with control therapy, PD-1 plus chem, PD-1 inhibitor plus chemotherapy combination therapy; G1–5, grades 1–5. **(B)** Forest plot analysis of cardiotoxicity in patients treated with CTLA-4/PD-L1 plus chemotherapy combination therapy compared with control therapy, CTLA-4 plus chem, CTLA-4 plus chemotherapy combination therapy; PD-L1 plus chem, PD-L1 plus chemotherapy combination therapy; G1–5, grades 1–5. **(C)** Forest plot analysis of cardiotoxicity in patients treated with PD-1 plus CTLA-4 combination therapy compared with control therapy: nivo plus ipili, PD-1 inhibitor (nivolumab) plus CTLA-4 inhibitor (ipilimumab) combination therapies; G1–5, grades 1–5.

As shown in **Figure 4**, PD-1 inhibitor plus chemotherapy show significant increase in grades 1–5 myocardial disease (OR 5.09, 95% CI: 1.11–23.32, p = 1.000), compared with chemotherapy alone. In contrast, CTLA-4 inhibitor (ipilimumab) plus chemotherapy showed no significant increase in grades 1–5 arrhythmology (OR 1.29, 95% CI: 0.47–3.57, p = 0.685), grades 1–5 cardiac failure (OR 1.43, 95% CI: 0.14–14.46, p = 0.124), grades 1–5 coronary artery disease (OR 0.86, 95% CI: 0.29–2.61, p = 0.623), or grades 1–5 pericardial disease (OR 0.89, 95% CI: 0.22–3.60, p = 0.420). (**Figure 4**)

Compared with chemotherapy and PD-1 inhibitor (nivolumab) or CTLA-4 inhibitor (ipilimumab), PD-1 inhibitor(nivolumab) plus CTLA-4 inhibitor (ipilimumab) combined therapy showed significant increase in grades 1–5 arrhythmology only (OR 2.49, 95% CI: 1.30–4.78, p = 0.289). Nevertheless, these ICIs did not show difference from chemotherapy in grades 1–5 cardiac failure (OR 0.50, 95% CI: 0.15–1.71, p = 0.489), grades 1–5 coronary artery disease (OR 1.79, 95% CI: 0.65–4.94, p = 0.791), grades 1–5 pericardial disease (OR 3.00, 95% CI: 0.31–28.92, p = 0.998), grades 1–5 cardiac arrest (OR 1.33, 95% CI: 0.30–5.96, p = 0.335), or grades 1–5 myocardial disease (OR 2.98, 95% CI: 0.47–18.95, p = 1.000). (**Figure 4**).

### Risk of Cardiotoxicity in Avelumab, Ipilimumab, Pembrolizumab and Nivolumab, Durvalumab

As shown in **eFigure 2**, there was no significant difference between PD-L1 inhibitor (avelumab) and chemotherapy in risk of in grades 1–5 arrhythmology (OR 2.82, 95% CI: 0.44–17.95, p = 0.992), grades 1–5 cardiac failure (OR 2.80, 95% CI: 0.29–27.04), grades 1–5 coronary artery disease (OR 1.40, 95% CI: 0.23–8.40), grades 1–5 pericardial disease (OR 8.45, 95% CI: 0.45–157.41), grades 1–5 cardiac arrest (OR 4.67, 95% CI: 0.22–97.56), or grades 1–5 myocardial disease (OR 4.67, 95% CI: 0.22–97.56).

Similarly, another PD-L1 inhibitor (durvalumab) did not show significant difference from chemotherapy in terms of grades 1–5 arrhythmology (OR 3.02, 95% CI: 0.36–25.22), grades 1–5 cardiac failure (OR 6.58, 95% CI: 0.37–117.24), grades 1–5 coronary artery disease (OR 9.67, 95% CI: 0.56–166.93), and grades 1–5 myocardial disease (OR 0.50, 95% CI: 0.03–7.98) (**eFigure 3**).

Likewise, compared with chemotherapy, CTLA-4 inhibitor (ipilimumab) did not show significant increase in risk of grades 1–5 arrhythmology (OR 1.28, 95% CI: 0.52–3.13, p = 0.200), grades 1–5 cardiac failure (OR 0.80, 95% CI: 0.27–2.16, p = 0.551), grades 1–5 coronary artery disease (OR 2.02, 95% CI: 0.61–6.71, p = 1.000), grades 1–5 pericardial disease (OR 0.54, 95% CI: 0.02–14.31, p = 0.130), or grades 1–5 cardiac arrest (OR 1.58, 95% CI: 0.61–4.11, p = 0.602) (**eFigure 4**).

Also, PD-1 inhibitors (pembrolizumab, nivolumab) show no significant increase of any risk compared with chemotherapy (**eFigures 5**, **6**).

### Risk of Cardiotoxicity in Different Doses/Cancer Types

As shown in **eFigure 7**, compared with a dose of 3 mg/kg q3w, a higher dose (10 mg/kg q3w) of CTLA-4 inhibitor (ipilimumab) showed no significant increase of risks in grades 1–5 arrhythmology (OR 13.15, 95% CI: 0.74–234.20). A dose of nivolumab 3 mg/kg plus ipilimumab 1 mg/kg also showed no significant increase of risks in grades 1–5 coronary artery disease (OR 7.04, 95% CI: 0.36–137.28) compared with a dose of ipilimumab 3 mg/kg plus nivolumab 1 mg/kg.

Similarly, compared with a dose of 10 mg/kg q2w, a dose of 10mg/kg q3w PD-1 inhibitor (pembrolizumab) did not show significantly increase risks of grades 1–5 cardiac failure (OR 0.21, 95% CI: 0.02–1.85).

In lung cancer and melanoma, there was no significant differences between ICIs and chemotherapy in risks of in grades 1–5 arrhythmology (OR 0.95, 95% CI: 0.50–1.81, p = 0.366; OR 1.29, 95% CI: 0.52–3.19, p = 0.467), grades 1–5 cardiac failure (OR 0.64, 95% CI: 0.30–1.36, p = 0.461; OR 3.84, 95% CI: 0.70–21.02, p = 0.431), grades 1–5 coronary artery disease (OR 1.24, 95% CI: 0.49–3.17, p = 0.347;OR 0.77, 95% CI: 0.22–2.66, p = 0.650), grades 1–5 pericardial disease (OR 0.86, 95% CI: 0.27–2.76, p = 0.296; OR 1.05, 95% CI: 0.15–7.20, p = 0.384), grades 1–5 cardiac arrest (OR 1.62, 95% CI: 0.65–4.00, p = 0.709) (**eFigures 8**, **9**).

### Quality Assessment and Publication Bias

Cochrane risk of bias tool was used to measure the quality of the included studies and the results were shown in **eFigures 1A**, **B**, which showed that the selected literatures are satisfactory. According to the Cochrane Handbook, the Q test and I2 statistics were employed to assess the heterogeneity among the RCTs. I2 values of lower than 30, 30–59, 60–75, and higher than 75% were classified as low, moderate, substantial, and considerable heterogeneity, respectively. There was a moderate heterogeneity (I2 values = 50%) in the PD-1 inhibitor compared with chemotherapy subgroup. Sensitivity analyses were also performed to assess the stability of the included studies. After excluding one study at a time, no significant difference in the results was found from the initial analysis. Moreover, the funnel plot, Egger and Begg test results showed a low risk of publication bias (Egger’s p-value = 0.155; Begg’s = 0.368) (**eFigures 10**, **11**).

## Discussion

Immunotherapy has been approved as a first-line treatment for metastatic melanoma and non-small cell lung cancer, a second-line treatment for Hodgkin’s lymphoma, head and neck cancer, renal cell cancer, and bladder cancer (31). Immunosuppressants inhibit tumor growth and metastasis by blocking the immune escape of tumor cells, which inevitably expose patients to different degrees of adverse events, and cardiotoxic-related adverse events are relatively infrequent (32). In 2014, adverse events related to cardiac toxicity were first reported by Heery et al. (33) since then, more and more cardiac toxicity related adverse events have been found during immunotherapy. Cardiac toxicity from immunotherapy is diversified, covering almost all parts of the heart. The most common adverse events are myocarditis and heart failure, which could lead to severe consequences including death. In a study of 964 patients by Mahmood et al., about 1.14% of patients developed myocarditis, and 0.52% experienced severe cardiovascular adverse events (e.g. complete heart block cardiac arrest) (32). A 2017 study on immunotherapy for non-small cell lung cancer found that the incidences of heart failure, cardiopulmonary arrest and myocardial infarction are 2, 1 and 1%, respectively, all of which were severe cardiovascular events (34).

This study is thus far the most comprehensive report on the impacts of immunotherapy on the heart, where all cardiotoxic-related adverse reactions were covered. The purpose of the study is to provide guidelines for the applications of immunosuppressants to clinical practice, which did not find significant differences in cardiac toxicity between many immunotherapies and chemotherapy. However, the meta-analysis showed that, compared with chemotherapy and PD-1 inhibitor (nivolumab) or CTLA-4 inhibitor (ipilimumab), PD-1 inhibitor (nivolumab) plus CTLA-4 inhibitor (ipilimumab) combined therapies showed significant increase in grades 1–5 arrhythmology (OR 2.49, 95% CI: 1.30–4.78, p = 0.006) and grade 5 arrhythmology (OR 3.90, 95% CI: 1.06–14.06, p = 0.04). Additionally, PD-1 inhibitor plus chemotherapy lead to more grades 1–5 myocardial disease (OR 5.09, 95% CI: 1.11–23.32, p = 0.04) than chemotherapy alone.

Consistent with our findings, some other existing studies showed that the combined treatment of nivolumab and ipilimumab has a high risk of heart-related adverse events. A phase one study of untreated melanoma showed that at least one fatal ventricular arrhythmia occurred in the early nivolumab and ipilimumab combined treatment group, and the probability of arrhythmia was higher (2.12%) than that of ipilimumab alone (0.32%). A phase three study included in our meta-analysis found that 16.3% of patients in the nivolumab group had grade 3 or grade 4 treatment-related adverse events while the number was 27.3 and 55.0% in the ipilimumab and combined group of nivolumab with ipilimumab, respectively. Meanwhile, the incidence of at least one severe arrhythmia in the combined group (1.6%) was even greater than that in the single-drug group (0.32%). The European medicines agency’s Opdivo public evaluation report revealed the incidence of heart-related adverse events in combination treatment of nivolumab and ipilimumab were: tachycardia (1.3%), arrhythmia (0.4%), and atrial fibrillation (0.2%) (35). Statistics also indicated that when ipilimumab and nivolumab were used simultaneously, the incidence of IrAEs can increase by 96%, and chances of grade 3 or grade 4 adverse events grow by 55%. All these events typically occur in the first 15 weeks of the treatment cycle and require less than 9 weeks of remission. Consequently, the combination of ipilimumab and nivolumab is currently inappropriate for 40% of patients.

In one case study, following three injections of combined immunotherapy (nivolumab and ipilimumab), a patient with left ventricular dysfunction developed heart failure symptoms where the left ventricular ejection fraction was dropped from 50 to 15% (36). The patient was diagnosed as immune-related myocarditis after biopsy. Our meta-analysis also found that most severe (including fatal) adverse events were almost all related to myocarditis. Fatal myocarditis has an incidence of 0.32% in the phase three study we analyzed. Bristol-Myers Squibb also evaluated the safety of its ipilimumab and nivolumab, and the data showed that ipilimumab and nivolumab combined treatment had a higher incidence of myocarditis (0.27%) than nivolumab monotherapy (37). In a clinical trial of combined therapy with ipilimumab and nivolumab, two fatal myocarditis cases occurred. Biopsy revealed infiltration of CD4+T, CD8+T cells and macrophages along with rhabdomyolysis in the myocardial tissue of these two patients (38). Furthermore, patients may experience chest pain, shortness of breath, fatigue, palpitations, edema of the lower limbs, or syncope. For patients without heart risk factors yet only hypertension history, the adverse reactions might be diagnosed as autoimmune-related myocarditis. Norwood et al. reported a myocarditis case 17 days after receiving ipilimumab plus nivolumab combined therapy. The patient’s Troponin I levels were 13 times higher than the normal, and myocardial biopsy revealed fibrosis with inflammation infiltrated by CD8+T cells. Myocarditis is also considered to be the earliest cardiotoxic reaction, occurring on average 17 days after initial treatment (39).

In addition, our study reported fatal myocarditis cases in patients with two other ICIs, namely, PD-L1 (avelumab) chemotherapy group (OR 2.79, 95% CI: 0.11–68.79, p = 0.53) and CTLA-4 (ipilimumab) chemotherapy group (OR 3.03, 95% CI: 0.12–74.46, p = 0.50). In total, patients with myocarditis treated with ICIs were 11 times more likely to develop myocarditis, compared with those treated without ICIs. Among the 964 patients treated by ICI at Massachusetts general hospital between 2013 and 2017, 1.14% (11 patients) developed myocarditis. A study on ipilimumab treatment (10 mg q3w) of a large dose also showed a fatal myocarditis following the treatment of the single drug (29). Myocarditis is the first adverse immune reaction that affected about 50% of patients. These patients often have no risk factors for heart disease before treatments, and 46% of them develop severe cardiovascular sequelae after cancer treatments (32). Corticosteroid medications can help to alleviate symptoms of most patients with nonfatal myocarditis.

The underlying physiological mechanism of myocardial injury caused by ICIs treatment has not been documented. In several cardiac biopsies of fatal myocarditis patients, the cardiac tissue was found to be infiltrated with CD4+/CD8+T lymphocytes and a few number of macrophages. Tarrio et al. found that myocardial injury was caused by PD-1-CD4+ T cells and PD-1-CD8+ T cells, and both T cell subsets needed PD-1 to maintain their tolerance to the components of the myocardium itself (40). Studies have found that the consumption of PD-1 can lead to impaired systolic function of the heart, leading to severe myocarditis and congestive heart failure. Lichtman et al. induced myocarditis by cytotoxic T cells and found that the genetic loss of PD-1 ligand, PD-L1 and PD-L2, as well as the treatment of PD-L1 inhibitors, can make transient myocarditis develop into a fatal disease. This demonstrated that PD-1 protects cardiomyocytes from their own T-lymphocytes. Another study in a mouse model found that the PD-1 knockout mice had the potential to develop spontaneous lupus arthritis and glomerulonephritis (41). In a preclinical model of Murphy Roths rats, downregulation of PD-L1 also leads to fatal autoimmune myocarditis. In cancer treatments, ICIs indirectly inhibits the protective effect of PD-1 and PD-L1 on their own cardiac tissue (42).

CTLA-4 has also been found to be closely related with cardiac homeostasis in mouse models, and the absence of CTLA-4 can lead to fatal myocarditis and pancreatitis. In the transplant response, blocking CTLA-4 can accelerate the acute rejection of heart transplantation. In a study by the German heart failure network, patients in the G/G genotype variant of Thr17Ala CTLA-4 group suffered higher risk of dilated cardiomyopathy (14.7%) than the control group (7%), suggesting that uncontrolled activation of T cells may promote tissue damage in the heart. Lichtman et al. found that CTLA-4 plays a vital role in regulating the functions of CD8+T cells in myocarditis, and absence of CTLA-4 increases the cardiac infiltration of CD8 + T cells driven by IL-12 (43). Initial T lymphocyte activation requires dendritic cells (DC) to present antigens to T cells through interaction of T cell receptors (TCR) and the major histocompatibility complexes (MHC). CTLA-4 regulates T cell activation as a transmissive inhibitory signal. The primary mechanism of ICIs is to release the inhibition of T cells by tumor cells. If the inhibition of T cells by cardiomyocytes is also blocked, the over-activated T cells may cause damage to the myocardium. CTLA-4 can competitively bind to CD80 (B7-2)/CD86 (B7-2) on antigen presenting cells (APCs) in the body, inhibit the activation of CD28, down-regulate the activity of helper CD4+T cell but enhance the activity of regulatory T cells (Treg) to achieve immunosuppression. Blocking CTLA-4 production can promote the proliferation and infiltration of CD4+T and CD8+T cells in the heart and the occurrence of myocarditis. PD-1 regulates the tolerance of myocardial tissue to the invasion of T lymphocytes, while CTLA-4 inhibits the over-activation of T lymphocytes. When used to treat the immunosuppressive effect of tumor, ICIs were thus inevitably cause immune abnormalities in the myocardium, leading to the occurrence of autoimmune myocarditis.

Currently, no standard management guidelines on ICI-related cardiotoxicity have been established, due to its low incidence and limited data on its manifestation, diagnosis, therapy, and outcomes (44). However, a prospective assessment of potential cardiotoxicity is warranted. From the clinical perspective, there are many potential risk factors, such as autoimmune diseases, pre-existing heart disease. Some studies have found that patients with underlying autoimmune diseases may be more susceptible to cardiotoxicity. For example, patients with systemic autoimmune diseases are more likely to develop subclinical myocarditis than patients without.^3^This suggests that a patient’s autoimmune status needs to be evaluated before initiating ICI treatment. Most of the patients with autoimmune cardiotoxicity had pre-existing heart disease or peripheral artery disease (45). In addition, individual differences in intestinal microbiota represent another source of heterogeneity in the efficacy of cancer immunotherapy and the toxicity of ICIs (46). The study of intestinal microbiota and the cardiotoxicity of ICIs is also of great clinical significance. From the genetic perspective, omics data can help us find biomarkers associated with cardiotoxicity. In glioma, multi-layer network biomarkers found by single-cell RNA sequencing technology can be applied to the prognosis and adverse reactions of tumors, and the prediction accuracy is better than that of traditional genetic biomarkers (47, 48). This method is novel and available, and may help us to find more suitable biomarkers for early prediction of cardiac toxicity.

In order to manage the cardiotoxicity associated with immunotherapy, we recommend that ECG and cardiac biomarkers should be monitored prior to the start of treatment to obtain baseline treatment information, and clinicians need to be vigilant for this complication.

## Limitations

Our meta-analysis examined the adverse cardiac effects of ICIs.

But this meta-analysis has limitations. Because of a small number yet diverse heart diseases reported, we have grouped these reactions according to internal medicine guidelines, which may omit details of specific heart diseases. Moreover, the clustering of research may result in the analysis inaccuracy and some subjective errors.

## Conclusions

In summary, our meta-analysis has clearly demonstrated that, compared with chemotherapy, 1) PD-1 inhibitor (nivolumab) or CTLA-4 inhibitor (ipilimumab) showed significant increase in grades 1–5 arrhythmology and grade 5 arrhythmology; 2) PD-1 inhibitor plus chemotherapy can significantly increase risks of grades 1–5 myocardial disease. According to the existing research, ICIs does cause cardiac adverse reactions when treating cancer patients. Therefore, it is very important for patients with heart disease history or/and high-risk factors to choose the appropriate program and dosage. A more heart friendly immunotherapy treatment is of high value in future research.

## Data Availability Statement

The datasets presented in this study can be found in online repositories. The names of the repository/repositories and accession number(s) can be found in the article/**Supplementary Material**.

## Author Contributions

JH and RT had access to all the data included in the study and are responsible for the completeness of the data and the accuracy of our analysis. YM and HZ helped to design the study. JH and XM contributed to the statistical analysis and the revision of this manuscript. QS and BC approved the final manuscript. All authors contributed to the article and approved the submitted version.

## Funding

This work was supported by Beijing Natural Science Foundation Program and Scientific Research Key Program of Beijing Municipal Commission of Education (KZ202010025047) and Training Program of the Major Research Plan of the National Natural Science Foundation of China (92046015).

## Conflict of Interest

The authors declare that the research was conducted in the absence of any commercial or financial relationships that could be construed as a potential conflict of interest.
