# Supplementary material for: Risk of Cardiac Adverse Events in Patients Treated With Immune Checkpoint Inhibitor Regimens: A Systematic Review and Meta-Analysis
Source: Front Oncol. 2021 May 27;11:645245. doi: 10.3389/fonc.2021.645245 (PMC8190385; doi:10.3389/fonc.2021.645245)
Supplement: Supplementary file 1 [file DataSheet_1.doc]

**eFigure 1a. Quality assessment for studies(Risk of bias graph)**

**
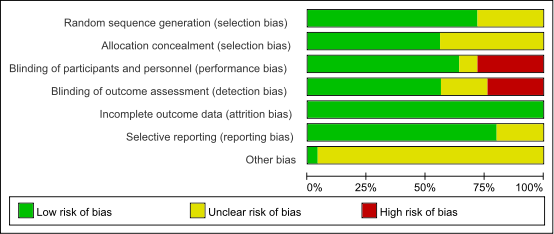
**

**eFigure 1b. Quality assessment for studies(Risk of bias summary)**

**
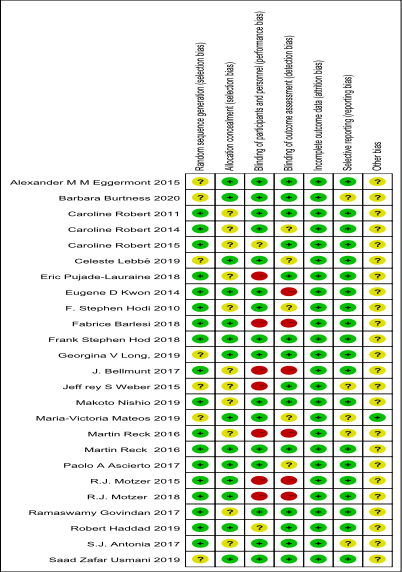
**

**eFigure 2.Forest plot analysis of cardiotoxicity in patients treated with PD-L1 inhibitor(avelumab)；G1–5: grade1–5.**

**
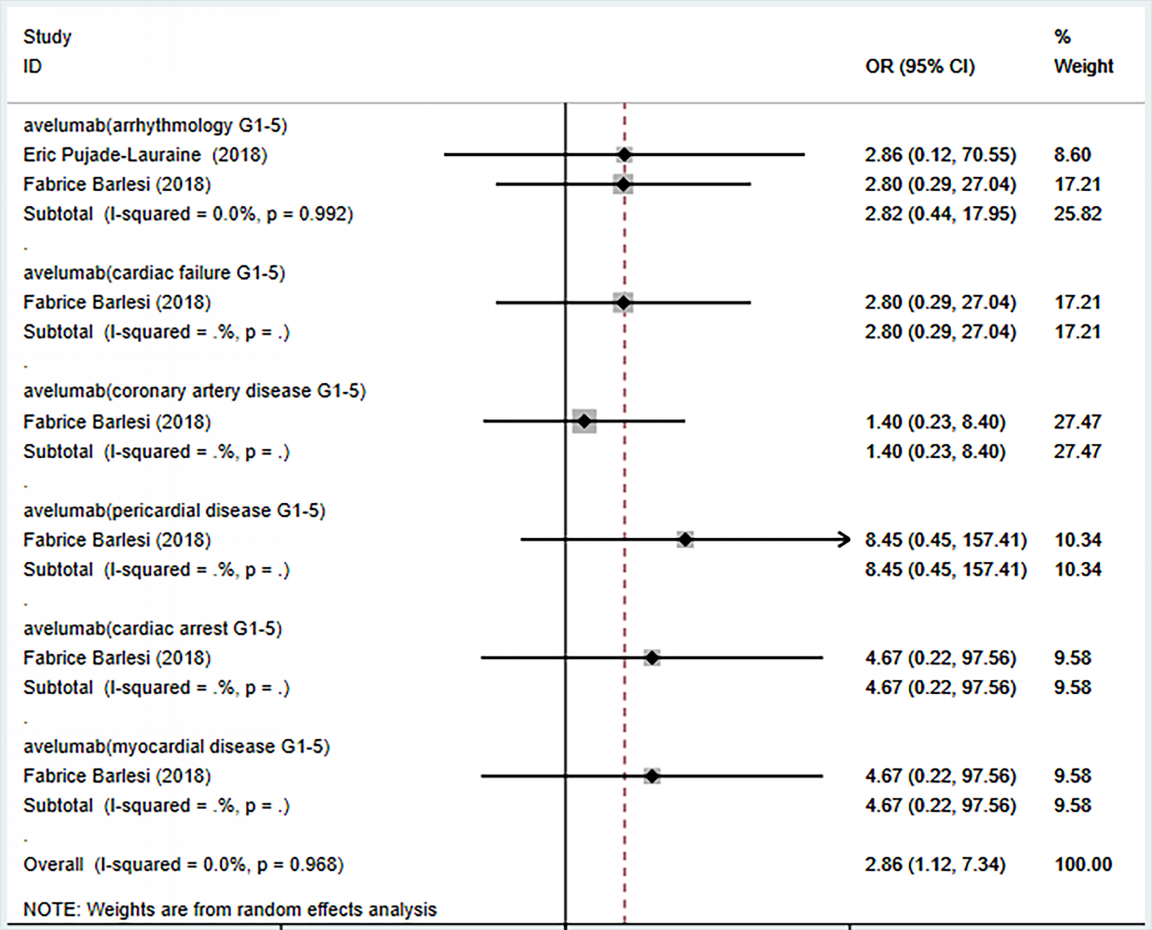
**

**eFigure 3.Forest plot analysis of cardiotoxicity in patients treated with PD-L1 inhibitor(durvalumab)；G1–5: grade1–5.**

**
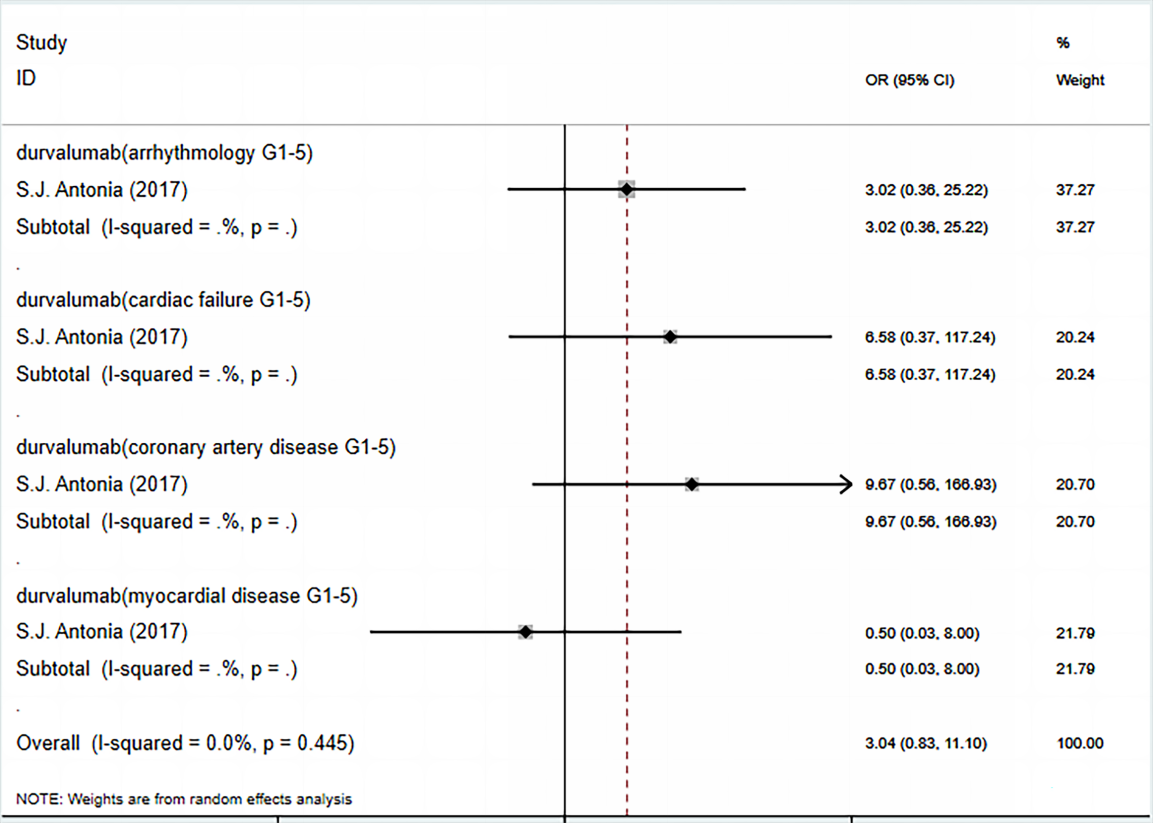
**

**eFigure 4.Forest plot analysis of cardiotoxicity in patients treated with CTLA-4 inhibitor(ipilimumab)；G1–5: grade1–5.**

**
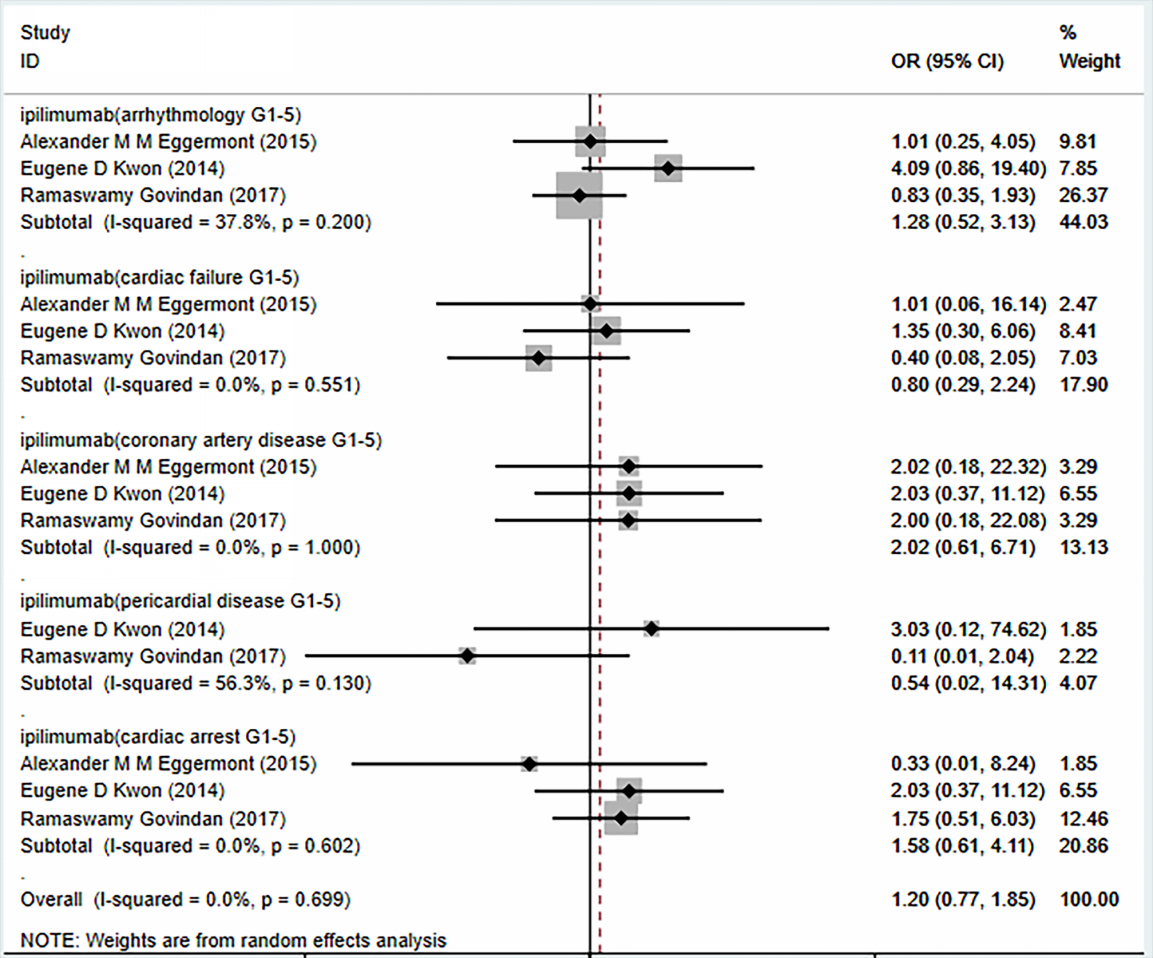
**

**eFigure 5.Forest plot analysis of cardiotoxicity in patients treated with PD-1 inhibitor(pembrolizumab)；G1–5: grade1–5.**

**
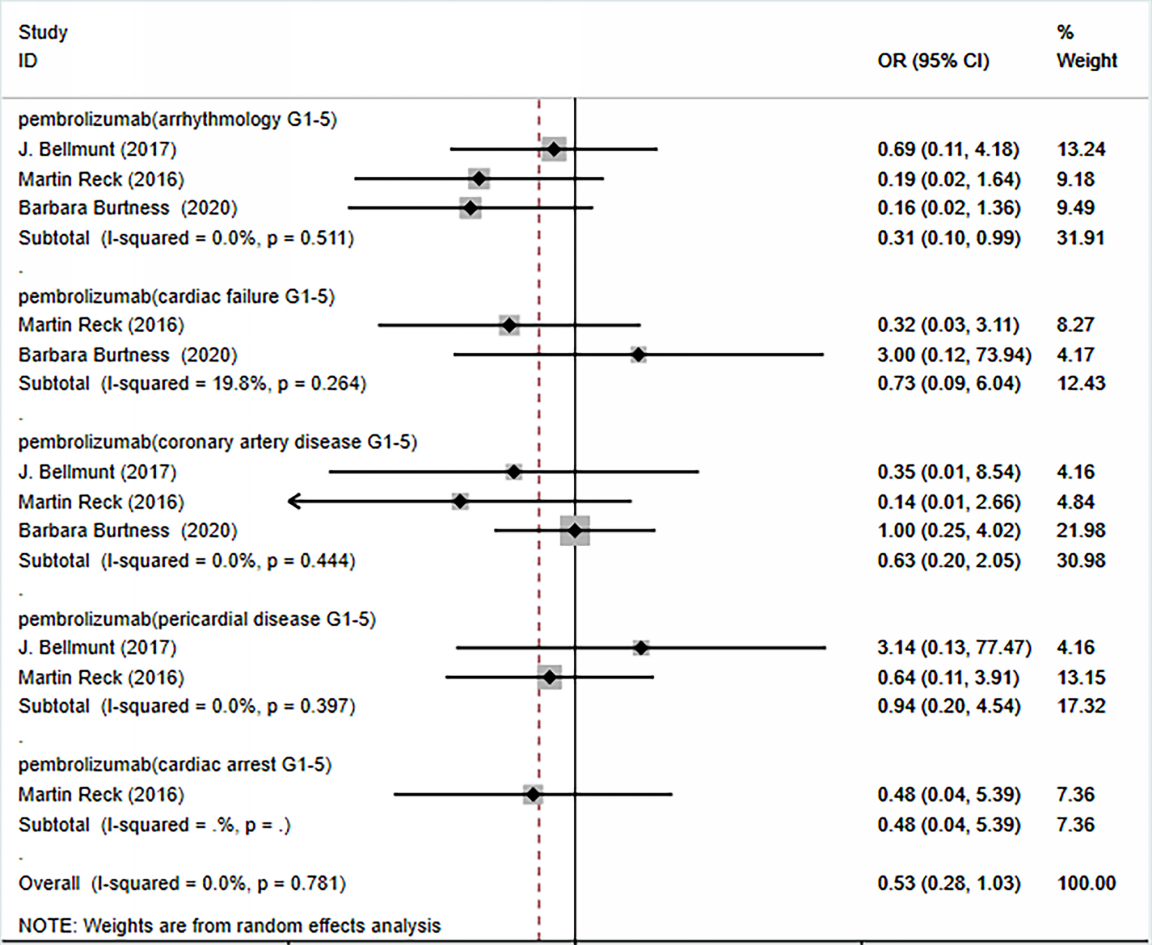
**

**eFigure 6.Forest plot analysis of cardiotoxicity in patients treated with PD-1 inhibitor(nivolumab)；G1–5: grade1–5.**

**
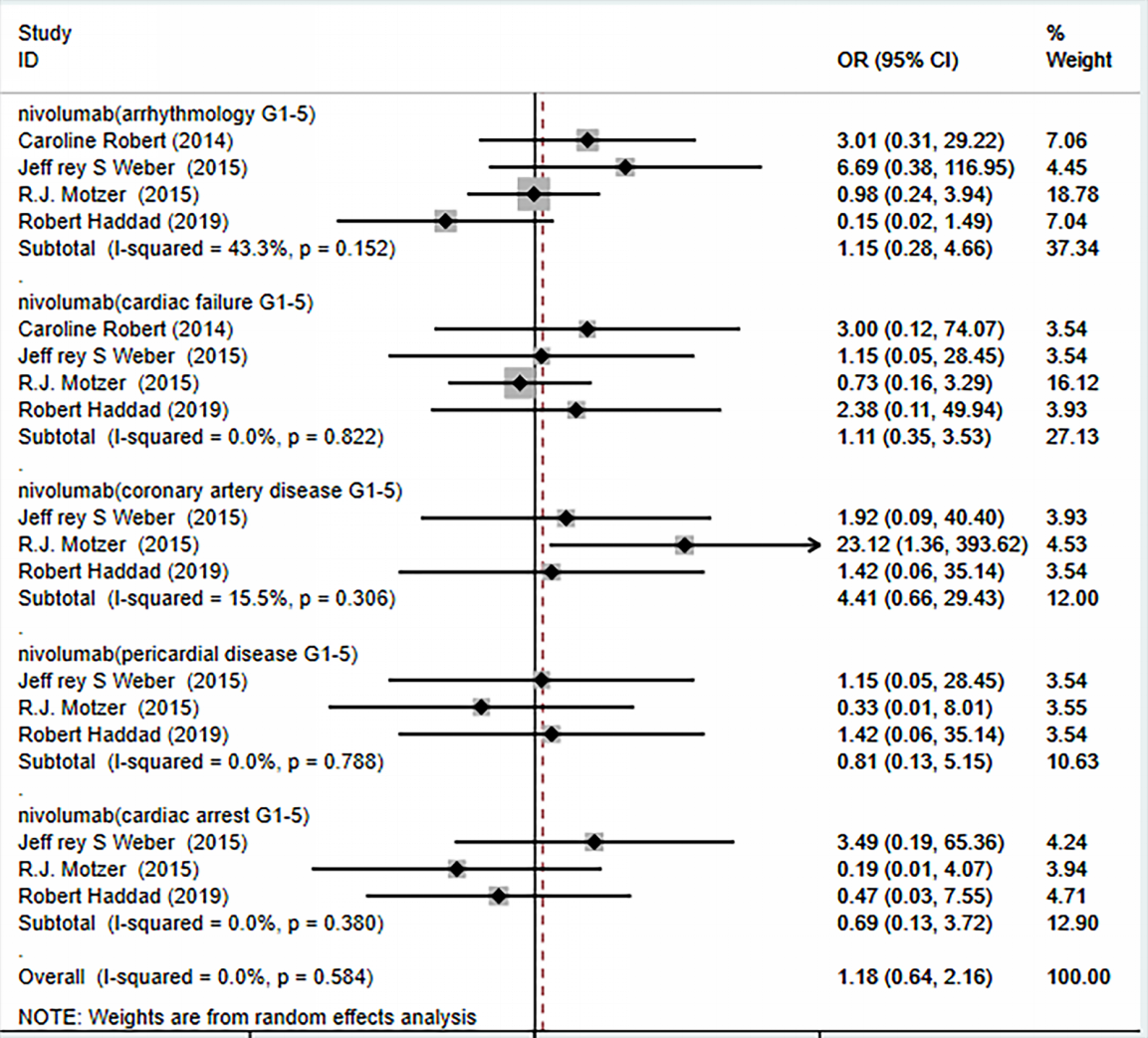
**
