# Supplementary material for: Risk of Cardiac Adverse Events in Patients Treated With Immune Checkpoint Inhibitor Regimens: A Systematic Review and Meta-Analysis
Source: Front Oncol. 2021 May 27;11:645245. doi: 10.3389/fonc.2021.645245 (PMC8190385; doi:10.3389/fonc.2021.645245)
Supplement: Supplementary file 2 [file DataSheet_2.doc]

**eFigure 7.Forest plot analysis of cardiotoxicity in patients treated with different doses:ipili 10 mg/kg q3w vs 3 mg/kg q3w:a dose of CTLA-4 inhibitor(ipilimumab) of 10 mg/kg q3w compared with a dose of 3 mg/kg q3w;nivo plus ipili:nivolumab 3 mg/kg plus ipilimumab 1 mg/kg vs ipilimumab 3 mg/kg plus nivolumab 1 mg/kg ;pembroli 10mg/kg q2w vs 10mg/kg q3w:a dose of PD-1 inhibitor(pembrolizumab) of 10mg/kg q2w compared with a dose of 10mg/kg q3w;G1–5: grade1–5.**

**
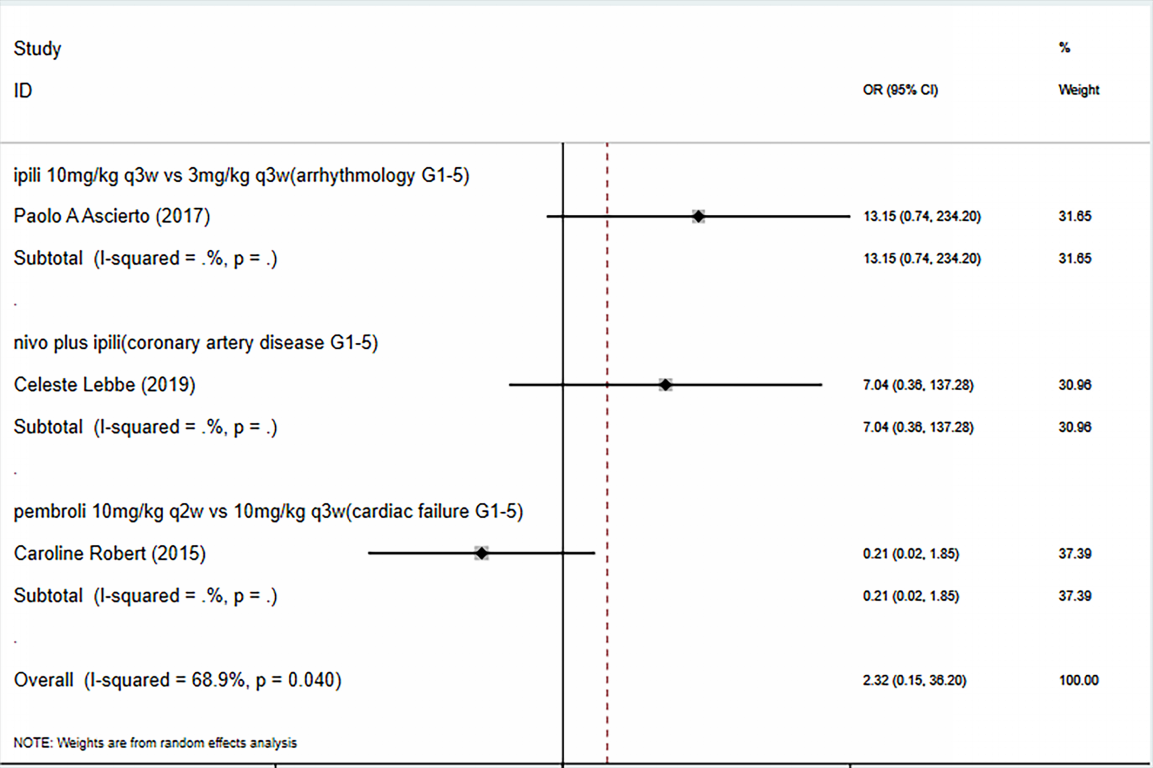
**

**eFigure 8.Forest plot analysis of cardiotoxicity in patients treated with ICIs in lung cancer**

**
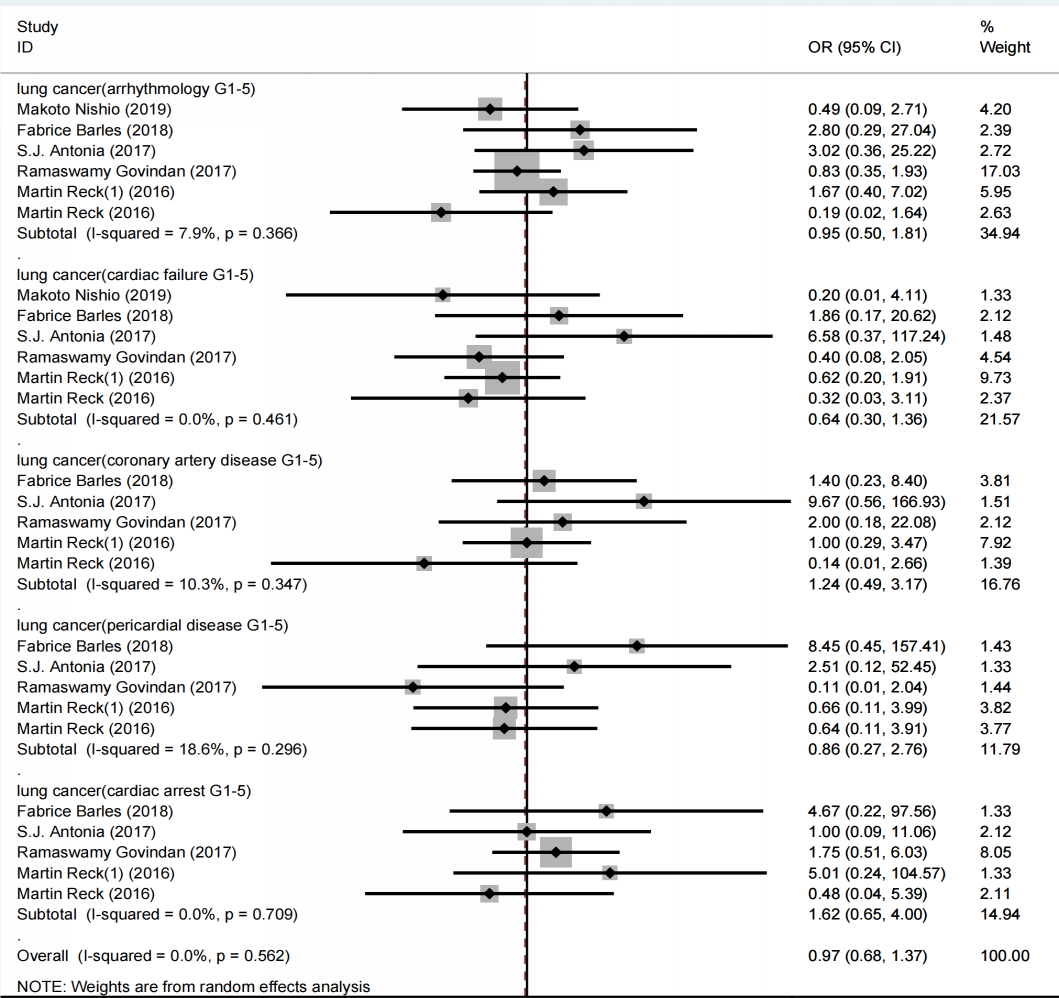
**

**eFigure 9.Forest plot analysis of cardiotoxicity in patients treated with ICIs in melanoma**

**
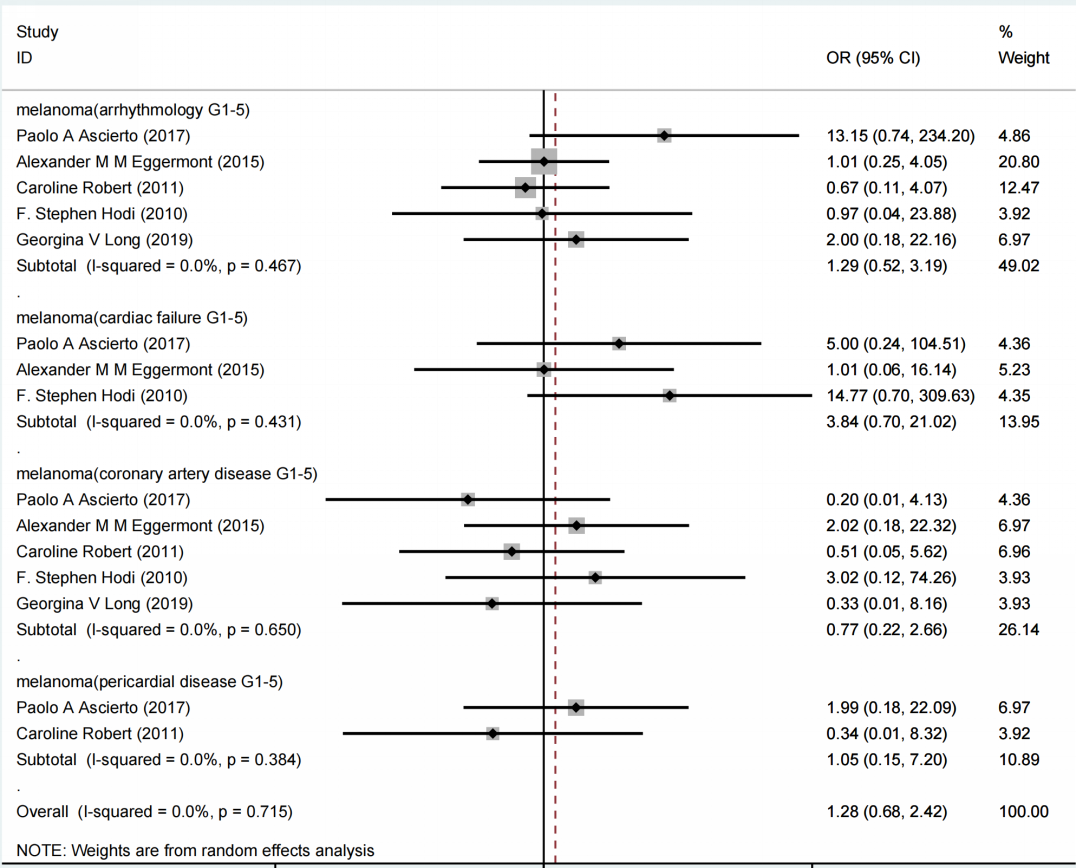
**

**eFigure 10.Results of publication bias（ funnel plot）**

**eFigure 11.Results of publication bias（Egger’s Test）**
